# The Epidemiological Pattern, Resistance Characteristics and Clinical Outcome of *Enterobacter cloacae*: Recent Updates and Impact of COVID-19 Pandemic

**DOI:** 10.3390/healthcare11030312

**Published:** 2023-01-19

**Authors:** Taghreed A. Hafiz, Alaa Albloshi, Ohoud S. Alhumaidan, Murad A. Mubaraki, Ahmed S. Alyami, Reem Alrashoudi, Mona A. Alrabiah, Fawzia Alotaibi

**Affiliations:** 1 Clinical Laboratory Sciences Department, College of Applied Medical Sciences, King Saud University, Riyadh 12372, Saudi Arabia; 2 Pathology and Clinical Laboratory Medicine, King Fahad Medical City, Riyadh 11525, Saudi Arabia; 3 Microbiology and Immunology Department, King Khaled University Hospital, Riyadh 12372, Saudi Arabia; 4 Pathology Department, College of Medicine, King Saud University, Riyadh 12372, Saudi Arabia

**Keywords:** *Enterobacter cloacae*, respiratory, ICU, nosocomial infection, MDR, PDR, COVID-19

## Abstract

Objectives: *E. cloacae* is an opportunistic organism that causes serious infections, particularly in immuno-compromised and hospitalized patients, along with the emergence of resistance traits. The COVID-19 pandemic has impacted the epidemiological pattern and resistance traits of *E. cloacae* infections as well as those of other bacteria. The study aims to assess the epidemiological patterns, resistance characteristics and clinical outcomes of *E. cloacae* in Saudi Arabia and the impact of the COVID-19 pandemic. Methods: King Fahad Medical City in Riyadh provided the data between January 2019 and December 2021 for the retrospective study of 638 isolates of *E. cloacae*. The clinical outcome of an *E. cloacae* infection was also determined by collecting and statistically analyzing the clinical records of 153 ICU patients. Results: The total percentage of resistant *E. cloacae* isolates decreased from 48.36% in 2019 to 38% in 2020 and 37.6% in 2021. The overall mortality rate among ICU patients was 40.5%, with an adult age group having a substantial relative risk value of 1.37. Conclusion: *E. cloacae* is a prevalent nosocomial infection in which adult age is a significant risk factor for mortality. Moreover, this study emphasizes the importance of comparing *E. cloacae* resistance trends before and throughout the pandemic period in order to better understand the bacteria’s behaviour.

## 1. Introduction

The Enterobacter genus is a member of the ESKAPE group (*Enterococcus faecium*, *Staphylococcus aureus*, *Klebsiella pneumoniae*, *Acinetobacter baumannii*, *Pseudomonas aeruginosa* and *Enterobacter* species) which can be considered as one of the main causes of the resistant nosocomial infections [1,2]. As a result of their ability to adapt to antimicrobial agents and opportunistic nature, in clinical settings, *Enterobacter cloacae*, *E. aerogenes* and *E. hormaechei* are the most frequently isolated species of *Enterobacter*, especially in immunocompromised patients and those in intensive care units (ICU) [3].

*E. cloacae* species is a commensal Gram-negative bacterium of the human gastrointestinal tract [4,5]. However, it can lead to a variety of infections such as urinary tract infections (UTI), pneumonia, lung abscesses and sepsis [6]. Recently, *E. cloacae* has attracted attention as a nosocomial pathogen that is frequently associated with several outbreaks in hospitals [7,8]. In addition, the genetic variation or heterogenicity and biochemical diversity among *E. cloacae* has emerged as *Enterobacter cloacae* complex (ECC) [9,10].

The SARS-CoV-2, severe acute respiratory syndrome coronavirus 2, pandemic had an impact on every aspect of life in the world, including population, economics and health care [11,12]. In addition, co-infection rates (either fungal or bacterial) among COVID-19 patients varied with a range between 3.5% to 15.5% [13]. Although, anti-microbial resistance (AMR) is a major worldwide issue, a number of studies have reported that this issue has grown during the SARS-CoV-2 pandemic [14,15]. In order to provide new findings about MDR pathogens during the COVID-19 pandemic, the aim of this study is to evaluate the epidemiological pattern, resistance characteristics and the clinical outcome of *E. cloacae* infection in Saudi Arabia as well as the impact of the pandemic.

## 2. Materials and Methods

### 2.1. Study Design and Setting

A three-year retrospective study was carried out at King Fahad Medical City (KFMC), which has a capacity of 1200 beds in Riyadh, Saudi Arabia, from January 2019 to December 2021. A total of 638 *E. cloacae* isolates from 638 patients’ clinical samples were studied. This study also included the clinical records of 154 ICU inpatients.

### 2.2. Data Collection

A total of 638 *E. cloacae* specimens were collected from different sources, including urine (in & out catheter, indwelling catheter, and mid-stream urine), respiratory (endotracheal and sputum), blood (peripheral and central lines), and miscellaneous (wound, tissue, abscess, body fluid, and device). The following were the inclusion criteria: (A) age was classified as pediatric or adult. Pediatrics is divided into three subcategories: infants (age 1 year), children (ages 1 to 10 years), and adolescents (ages from 11 to 18 years). Furthermore, the adults were classified into four subcategories: group one (aged 19 to 44 years old), group two (aged 45 to 64 years old), group three (aged 65 to 84 years old), and group four (aged 85 and up); (B) the ward or clinic where the patient was conceded (outpatient clinics, emergency, ICU, and other wards); (C) the source and location/site of the sample; and (D) the bacterial resistance category, which involves susceptible, multi-drug resistant (MDR), extensive-drug resistant (XDR), and pan-drug resistant bacteria (PDR). Any isolate apart from *E. cloacae* was excluded from the study. Additionally, the clinical histories of patients admitted to the ICU, including both adult and pediatric patients, were obtained from the KFMC medical database. The clinical history included the following criteria: (1) chronic disease such as diabetes mellitus (DM), hypertension, renal disease, or malignancy; (2) exposure to carbapenem or other antibiotics in the past 14 to 30 days; (3) the presence of bacteremia or septicemia; (4) on mechanical ventilation or not; (5) co-infection, if present or not; (6) the presence of clinical symptoms such as fever, gastrointestinal tract (GIT) symptoms, or respiratory symptoms; (7) the presence of wound or urinary tract infection; (8) renal dialysis at isolation or not; (9) clinical outcomes for the patient and additional notes if present. 

### 2.3. E. cloacae Identification and Antimicrobial Susceptibility Testing

With the help of The BD Phoenix^TM^ automated microbiology system (Becton Dickinson Diagnostic Systems, Sparks, Md.) for full identification and sensitivity testing, all isolates are presumptively identified as *Enterobacter* species. Only patients with isolates that were positively identified as *E. cloacae* were included in the study. The following antibiotics were tested for antimicrobial sensitivity (AST): ampicillin (AMP), amoxicillin-clavulanate (AMC), piperacillin-tazobactam (TZP), imipenem (IPM), meropenem (MER), ertapenem (ETP), cephalothin (CEF), cefuroxime (CXM), ceftazidime (CTZ), cefoxitin (FOX), cefepime (CFP), cefotaxime (CTX), ceftriaxone (CRO), ciprofloxacin (CIP), levofloxacin (LVX), gentamicin (GM), amikacin (AMK), trimethoprim-sulfamethoxazole (TMP-SMX). The results were classified as susceptible (S), intermediate (I) and resistant (R). Confirmation of resistant isolates was performed by E-test. The *E. cloacae* isolates were categorized based on their resistance to antibiotics, according to International Consensus [16]. Results were interpreted and reported according to the 32nd Edition of the CLSI-M100 document. The classifications were as follows: (1) multi-drug resistant, indicating resistance to three or more classes of antibiotics; (2) extensive-drug resistant, indicating resistance to at least one agent in all but two or fewer antibiotic classes; and (3) pan-drug resistant, indicating resistance to all possible antibiotics. For those isolates that reported resistance to all the aforementioned antibiotics, AST was performed for Colistin antibiotic.

### 2.4. Statistical Analysis 

All data were analyzed using GraphPad prism statistical project version 9.3.1. General analysis was performed to generate representative graphs and charts for the following data: age groups, gender, ward/clinic, sample source, and sample site with the aid of a contingency graph and relevant parts of whole graphs. The resistance characteristics and distribution pattern of *E. cloacae* between 2019, 2020, and 2021 were compared by the contingency graph. In addition, more specific statistical analysis was performed for the clinical data of ICU patients and the descriptive data were expressed as an absolute number (n) and percentage. The *p*-value of <0.05 was considered statistically significant. 

### 2.5. Ethical Approval

The project was approved by King Fahad Medical City’s local ethical research committee. KFMC provided consent in accordance with ICH GCP guidelines and the ethical code of IRB log number: 21-426E. 

## 3. Results

### 3.1. Demographic, Clinical and Baseline Laboratory Distribution of Sample Size over a Three-Year Period between 2019 and 2021

**Figure 1:**
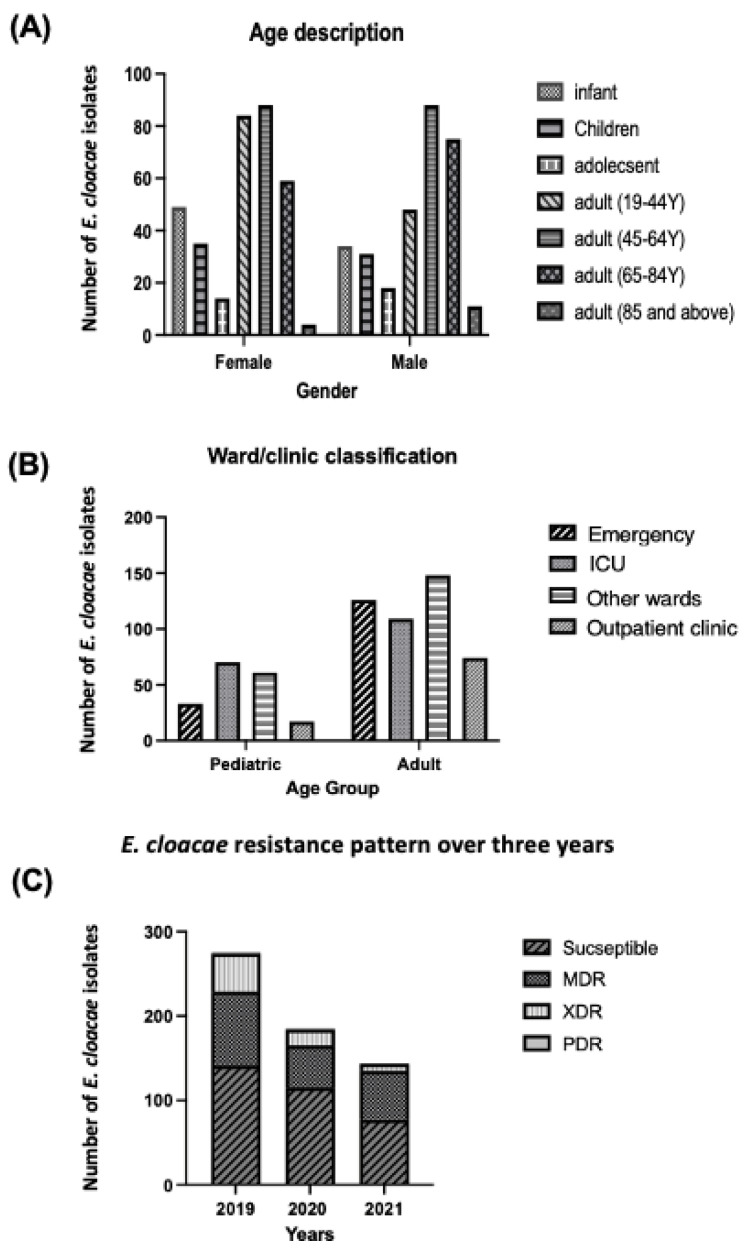
Demographic, clinical and baseline laboratory sample size distribution over a three-year period between (2019–2021). (**A**) Age description for male and female. (**B**) Ward/clinic description for pediatric and adult. (**C**) *E. cloacae* resistance pattern. MDR, XDR, PDR.

In the study period, a total of 638 items of data were collected (333 female and 305 male). Female data composed of 14.7% (infant), 10.5% (children), 4.2% (adolescent), 25.2% (adult 19–44Y), 26.4% (adult 45–64Y), 17.7% (adult 65–84Y) and 1.2% (adult of 85Y). Male data include 11% (infant), 10.2% (children), 6% (adolescent), 15.7% (adult 19–44Y), 29% (adult 45–64Y), 24.6% (adult 65–84Y) and 3.6% (adult of 85Y). as illustrated in (Figure 1A). As shown in Figure 1B, the wards and clinics were divided into pediatric and adult according to age and the results were demonstrated as follows: pediatric patients (emergency 18.2%, ICU 38.7%, other ward 33.7% and outpatient clinic 9.4%), adult patients (emergency 27.57%, ICU 24%, ward 32.4% and outpatient clinic 16.2%). Regarding the resistance pattern over the three years, this is shown in (Figure 1C). During 2019, 51.72% of the total 275 isolates were susceptible, 32% MDR, 16.36% XDR and 0.36% PDR. In 2020, 62.16% of the total 185 isolates were susceptible, 27.03% MDR, 10.27% XDR and 0.54% PDR. During 2021, 53.47% of the total 178 isolates were susceptible, 40.28% MDR, 5.56% XDR and 0.69% PDR. 

### 3.2. Demographic and Clinical Characteristics of ICU Patients

A statistical analysis of the clinical histories of 153 ICU patients is presented in Table 1. Infants are much more likely to be admitted to the ICU due to infection (51% vs. 13.2% and 6.5% for children and adolescents, respectively). Additionally, compared to other adult patient categories, admission of patients in the age range 65–84 is more common, with a rate of 52%. The prevalence of respiratory symptoms is 45.71% for female ICU patients and 60.24% for male patients. Notably, with a rate of 92.81%, the majority of ICU patients receive mechanical ventilation. Renal disease, diabetes, hypertension and cancer have a rate of 30.7%, 37.3%, 45.8% and 22.2% respectively, for chronic diseases associated with ICU patients (Table 1). 

### 3.3. Clinical Outcomes of ICU Patients Infected with E. cloacae 

The mortality rate among ICU patients (*n* = 135) with *E. cloacae* infection was 40.5%, comprising 62 of the 153 patients. Regarding the AST analysis, 64% of the clinical isolates were classified as susceptible, while 23%, 11.8% and 1.3% were identified as MDR, XDR and PDR, respectively (Table 2). Furthermore, applying univariate analysis for factors associated with mortality of ICU patients infected with *E. cloacae*, adult age group showed to be a significant factor associated with mortality (Fisher’s exact test; *p* ≤ 0.05) and relative risk (RR) of mortality was also significant (RR = 1.37 with CI95% 1.0656 to 1.7636, *p* value = 0.014).

### 3.4. Comparison among ICU and Non-ICU Patients over a Three-Year Period between (2019–2021)

In 2019, 48.36% of *E. cloacae* isolates were found to be resistant, compared to 38% and 37.6% of clinical isolates in 2020 and 2021, respectively. Furthermore, respiratory samples were statistically significant, with a *p*-value of (0.0001), because they were the most common specimen source among ICU patients, as opposed to non-ICU patients, where urine samples were statistically more frequent over a three-year period. In addition, the collection rate of miscellaneous samples among ICU and non-ICU patients was similar over the three years. However, the sampling rate among non-ICU patients is much higher than ICU patients (Table 3). 

### 3.5. Antimicrobial Susceptibility Pattern of E. cloacae Isolates 

Since *E. cloacae* is an AmpC β-lactamase producer, the AST results confirmed the resistance to penicillin (AMP and AMC) along with cephalosporins (CEF, CXM and FOX) with percentages of 100%, 99.84%, 99.84%, 79.31% and 96.5% respectively. *E. cloacae* was susceptible to Amikacin, AMK (98.6%), Levofloxacin, LVX (91%), Ertapenem, ETP (90.75%), Meropenem, MER (90.44%), Imipenem, IPM (89.18%), Gentamicin, GM (89.2%), Cefepime, CFP (80.6%) and Piperacillin-Tazobactam, TZP (79.15%) (Table 4). Regarding the *E. cloacae* PDR isolates, both isolates showed resistance to colistin.

## 4. Discussion

The clinical significance and genetic diversity of the *E. cloacae* complex (ECC) are well studied. However, little is known about the resistance, clinical features of *E. cloacae*-infected patients and the overall outcome of infection before and during the COVID-19 pandemic. The current study focused on these mentioned parameters for infected patients in ICU and non-ICU wards by analyzing the clinical and microbiological data.

In the current study, the three-year analysis from 2019 to 2021 included 638 isolates—333 female and 305 male. The majority (29%) of *E. cloacae* isolates were found in males between the ages of 45 and 64. A study from Colombia was done between 2011 and 2018 in five hospitals by Falco et al. (2021), which showed that 80% of KPC-producing *E. cloacae* complex were found in males (80%) and 29% were between the age of 41 and 60 [17]. In another study performed in Iran among 649 patients with positive Enterobacter from 2016 to 2018, 54.7% were male and 45.3% were female [18]. In China, a study conducted in a teaching hospital between 2015 and 2018, showed that 62% of the patients with *E. cloacae* were male [19]. 

Identification of *E. cloacae* sources in hospital and community settings is a crucial first step in preventing infection of those who are susceptible. In our study, most of the positive cases were isolated in the ICU (38.7%). This result was in agreement with other studies that found the majority of *E. cloacae* isolates were found in patients admitted to adult [18,20] and pediatric ICUs [3,20]. ICUs, adult and pediatric wards, and neonatal wards are among the hospital sections where *E. cloacae* infection outbreaks have been reported [21,22]. These findings could be attributed to the immune state of those individuals as well as the fact that *E. cloacae* is recognized as a human opportunistic pathogen in the healthcare setting. 

Our findings indicated that the prevalence of *E. cloacae* infection decreased in 2020 and 2021 compared to 2019 based on the resistance pattern of E. cloacae. The priority given to COVID-19 infected patients may have resulted in fewer patients being tested for E. cloacae. This result is similar to one from Hirabayashi et al., who found a decrease in the number of patients requiring bacterial isolation in 2020 compared to 2019, particularly in hospitals where COVID-19 patients are given priority [23]. 

All three of the resistant categories (MDR, XDR, and PDR) were found to be significantly higher in 2019 than in 2020 and 2021 with regard to the resistance type of E. cloacae. The reduction in the resistance rate of *E. cloacae* isolates in 2020 and 2021 could be due to the restricted hospital admission policy during the pandemic. In addition, COVID-19 vaccine decreased the rate of hospitalization. A published study in 2021 also showed that the rate of hospitalization was reduced to more than 70% after vaccination, especially for people aged 80 years or more [24]. 

The analysis of demographic and clinical characteristics of *E. cloacae* ICU patients indicated that infants were the most affected age group in the pediatric categories. This result is in agreement with several studies reporting an outbreak due to *E. cloacae* in neonatal ICUs [3,25,26,27]. Early gut colonization with the *E. cloacae* complex is common in preterm neonates [28]. As a result, ECC colonization can serve as a source of infection or transmission, especially among immunocompromised patients [25].

In the adult categories of ICU patients, those aged between 65 and 84 years are the most commonly diagnosed with *E. cloacae* (52%). This might be caused by the patient’s prolonged stay in the ICU, immunity status, age, gender and mechanical ventilation, all of which were considered to be risk factors for developing an infection with *E. cloacae* for both adults and pediatric groups [4,28]. The age group was found to have a significant effect on the outcomes among ICU patients infected with E. cloacae, with pediatric patients having a better outcome (alive, 42.86%) and adults having a significantly worse outcome (deceased, 77.42%), 1.37 times more likely to die than pediatric. 

In terms of *E. cloacae* specimen type, respiratory specimens were significantly greater among ICU patients over three years, but urine specimens were significant among non-ICU patients, reflecting the majority of ICU patients’ mechanical ventilation status. The need for mechanical ventilation and COVID-19 have previously been linked [29,30]. Furthermore, this could be related to *E. cloacae* co-infection with SARS-CoV-2, since studies in the United Arab Emirates [31], New York [32] and Italy [33] reported the prevalence of *E. cloacae* co-infection among COVID-19 patients. The type of co-infection among ICU patients in this study, however, was not available. Significantly, urinary tract infection was observed among ICU patients (42 out of 153). This is considered to be a risk factor associated with *E. cloacae* infection. In addition, several studies reported that the *E. cloacae* were frequently detected in cases of UTI, especially in renal transplant patients [34,35]. 

In the present study, *E. cloacae* was susceptible to several antibiotics and the most common were Amikacin, AMK (98.6%), Levofloxacin, LVX (91%), Ertapenem, ETP (90.75%), Meropenem, MER (90.44%), Imipenem, IPM (89.18%), Gentamicin, GM (89.2%), Cefepime, CFP (80.6%) and Piperacillin-Tazobactam, TZP (79.15%). According to published reports, the resistance of *E. cloacae* strains varies according to the year of testing [36]. As previously mentioned, the SARS-CoV-2 pandemic may have only a minor impact on the emergence of antimicrobial resistance and this could be because infection control measures such as hand washing, the use of personal protective equipment (PPE), and decontamination procedures have been increased to reduce infections among healthcare workers [37]. Finally, due to the lack of similar studies, it is extremely challenging to compare the current data for the resistant pattern with other studies [38,39]. 

## 5. Strengths and Limitations

The sample size of the study contains a variety of different sample sources with variable sites specifying the type of infection, which gives a clearer picture regarding the cause of *E. cloacae* infection. The limitation of the study is the small sample size: even of the total number of samples (638) is not considered low, a greater number would give us much more precise findings in regard to statistical analysis and the study included only the data collected from KFMC in Riyadh. In addition, this study sheds light on the clinical characteristics of *E. cloacae* during the pandemic, which guided us to understand more about the resistance behavior of the bacteria. Nevertheless, more studies must be performed to fully understand the effect of the COVID-19 pandemic on the resistance characteristics of *E. cloacae* bacteria.

## 6. Conclusions

In the end, during the COVID-19 pandemic, there was a decrease in *E. cloacae* resistance, while a rise in the number of *E. cloacae* respiratory samples among ICU patients revealed that *E. cloacae* is a common co-infection linked with mechanical ventilation and possibly SARS-CoV-2. This study stresses the necessity of comparing *E. cloacae* resistance patterns before and throughout the pandemic period to better understand the bacteria’s behavior.

## Figures and Tables

**Table 1:** Comparison of the demographic and clinical characteristics of ICU patients infected with *E. cloacae*.

Characteristics	Gender		
	Females *n* (%) (Total = 70)	Males *n* (%) (Total = 83)	*p* Value
Age group			
Infant	24 (34.29%)	14 (16.87%)	0.0152 *
Children	5 (7.14%)	5 (6.02%)	>0.9999
Adolescent	2 (2.86%)	3 (3.61%)	>0.9999
Adult (19–44Y)	11 (15.71%)	15 (18.07%)	0.8296
Adult (45–64Y)	8 (11.43%)	21 (25.30%)	0.0380 *
Adult (65–84Y)	19 (27.14%)	21 (25.30%)	0.8544
Adult (85Y and above)	1 (1.43%)	4 (4.82%)	0.3759
Mortality	32 (45.71%)	30 (36.14%)	0.2506
Clinical presentation/infection			
Co-infection	29 (41.43%)	22 (26.51%)	0.0595
Fever	20 (28.75%)	32 (38.55%)	0.2316
GIT manifestations	11 (15.71%)	15 (18.07%)	0.8296
Respiratory symptoms	32 (45.71%)	50 (60.24%)	0.0767
Renal dialysis	4 (5.71%)	8 (9.64%)	0.5480
Undergo mechanical ventilation	66 (94.29%)	76 (91.57%)	0.7550
Septicemia	18 (25.71%)	17 (20.48%)	0.4482
Bacteremia	11 (15.71%)	7 (8.43%)	0.2098
Septic shock	5 (7.14%)	11 (13.25%)	0.2915
UTI	18 (25.71%)	24 (28.92%)	0.7180
Wound infection	13 (18.57%)	15 (18.07%)	>0.9999
Associated chronic diseases			
Renal disease	18 (25.71%)	29 (34.94%)	0.2913
Diabetes	22 (31.43%)	35 (42.17%)	0.1836
Hypertension	25 (35.71%)	45 (54.22%)	0.0239 *
Malignancy	20 (28.57%)	14 (16.87%)	0.1177

The statistical significance difference between male and female patients is indicated by a (*) symbol (Fisher’s exact test; *p* ≤ 0.05). GIT (gastro-intestinal tract), UTI (urinary tract infection).

**Table 2:** Factors associated with mortality of ICU patients infected with *E. cloacae*.

Characteristics	Outcome		
	Deceased *n* (%) (Total = 62)	Alive *n* (%) (Total = 91)	*p* Value
Age group			
Pediatric	14 (22.58%)	39 (42.86%)	0.0101 *
Adult	48 (77.42%)	52 (57.14%)	0.0101 *
Source of specimen			
Blood samples	12 (19.35%)	17 (18.68%)	>0.9999
Respiratory samples	32 (51.61%)	47 (51.65%)	>0.9999
Urinary tract samples	5 (8.06%)	12 (13.19%)	0.4343
CSF samples	3 (4.84%)	1 (1.1%)	0.3040
Miscellaneous samples	10 (16.13%)	14 (15.4%)	>0.9999
Resistance categories			
Susceptible	33 (53.23%)	65 (71.43%)	0.0260 *
MDR	16 (25.81%)	19 (20.88%)	0.8202
XDR	11 (17.74%)	7 (7.69%)	0.0745
PDR	2 (3.23%)	0	0.1626

The statistical significance difference between Deceased and Alive patients is indicated by a (*) symbol (Fisher’s exact test; *p* ≤ 0.05). MDR (multi-drug resistance), XDR (Extensive-drug resistant), PDR (pan-drug resistant).

**Table 3:** Comparison among ICU and non-ICU patients over three years (2019–2021).

Characteristics	ICU *n* (%)	Non-ICU *n* (%)	*p* Value
Year of 2019	Total = 86	Total = 189	
Source of sample			
Blood samples	28 (32.6%)	38 (20.11%)	0.0326 *
Respiratory samples	29 (33.72%)	6 (3.51%)	<0.0001 ****
Urinary samples	13 (15.21%)	92 (48.7%)	<0.0001 ****
CSF samples	1 (1.16%)	4 (2.12%)	<0.9999
Miscellaneous samples	15 (17.4%)	49 (26%)	0.1655
Resistant category			
Susceptible	53 (61.6%)	88 (46.6%)	0.0267 *
MDR	16 (18.6%)	72 (38.1%)	0.0013 **
XDR	16 (18.6%)	29 (15.3%)	0.4878
PDR	No cases		
Year of 2020	Total = 46	Total = 139	
Type of sample			
Blood samples	8 (17.4%)	37 (26.6%)	0.2388
Respiratory samples	26 (56.5%)	14 (10.07%)	<0.0001 ****
Urinary samples	5 (10.8%)	42 (30.2%)	0.0104 *
CSF samples	1 (2.17%)	1 (0.72%)	0.4365
Miscellaneous samples	6 (13.04%)	45 (32.37%)	0.0128
Resistant category			
Susceptible	30 (65.2%)	85 (61.1%)	0.7264
MDR	11 (24%)	39 (28.06%)	0.7026
XDR	4 (21.05%)	15 (11%)	0.7866
PDR	1 (2.17%)	0	0.2486
Year of 2021	Total = 47	Total = 131	
Type of sample			
Blood samples	5 (10.64%)	30 (23%)	0.0872
Respiratory samples	33 (70.2%)	19 (14.5%)	<0.0001 ****
Urinary samples	1 (2.13%)	28 (21.4%)	0.0011 **
CSF samples	2 (4.26%)	1 (0.8%)	0.1708
Miscellaneous samples	6 (12.7%)	53 (40.5%)	0.0005 ***
Resistant category			
Susceptible	34 (72.3%)	77 (58.8%)	0.1159
MDR	12 (25.53%)	46 (35.1%)	0.2780
XDR	1 (2.13%)	7 (5.34%)	0.6829
PDR	0	1 (0.76%)	>0.9999

The statistical significance difference between Deceased and Alive patients is indicated by a (*) symbol and the number of * represents the strength of the significant difference (Fisher’s exact test; *p* ≤ 0.05). CSF (Cerebrospinal fluid).

**Table 4:** Antimicrobial susceptibility test results for *E. cloacae* isolates.

(Total = 638)			
	Susceptible %	Intermediate %	Resistant %
AMP	0	0	100
AMC	0.16	0	99.84
TZP	79.15	7.21	13.64
IPM	89.18	0.94	9.87
MER	90.44	0	9.56
ETP	90.75	0	9.25
CEF	0.16	0	99.84
CXM	15.2	5.5	79.31
CTZ	73.2	3.6	23.2
FOX	3.4	0	96.5
CFP	80.6	6.4	13
CTX	65.5	0.5	34
CRO	65.4	0.6	34
CIP	86.1	3.4	10.5
LVX	91	1.8	7.4
GM	89.2	0.16	10.7
AMK	98.6	0	1.4
TMP-SMX	77.1	0	22.8

AMP (Ampicillin), AMC (Amoxicillin-Clavulanate), TZP (Piperacillin-Tazobactam), IPM (Imipenem), MER (Meropenem), ETP (Ertapenem), CEF (Cephalothin), CXM (Cefuroxime), CTZ (Ceftazidime), FOX (Cefoxitin), CFP (cefepime), CTX (Cefotaxime), CRO (Ceftrixone), CIP (Ciprofloxacin), LVX (Levofloxacin), GM (Gentamicin), AMK (Amikacin), TMP-SMX (Trimethoprim-Sulfa-methaxazole).

## Data Availability

Data is stored in the KFMC institute data system and may be available to the general public upon special request.
